# Effects of the timing of administration of IgM- and IgA-enriched intravenous polyclonal immunoglobulins on the outcome of septic shock patients

**DOI:** 10.1186/s13613-018-0466-7

**Published:** 2018-12-10

**Authors:** Giorgio Berlot, Michele Claudio Vassallo, Nicola Busetto, Margarita Nieto Yabar, Tatiana Istrati, Silvia Baronio, Giada Quarantotto, Mattia Bixio, Giulia Barbati, Roberto Dattola, Irene Longo, Antonino Chillemi, Alice Scamperle, Fulvio Iscra, Ariella Tomasini

**Affiliations:** 1 Department of Anesthesia, Resuscitation and Pain Therapy, Cattinara Hospital, University of Trieste, Strada di Fiume 447, 34149 Trieste, Italy; 2 Department of Anesthesia and Intensive Care, San Martino Hospital, Largo Rosanna Benzi 10, 16132 Genoa, Italy; 3 Biostatistics Unit, Department of Medicine, Surgery and Health Sciences, Cattinara Hospital, University of Trieste, Strada di Fiume 447, 34149 Trieste, Italy

**Keywords:** Severe sepsis, Septic shock, Immunoglobulins, IgM, Multiple drug resistance, Intensive care unit

## Abstract

**Background:**

The administration of endovenous immunoglobulins in patients with septic shock could be beneficial and preparations enriched with IgA and IgM (ivIgGAM) seem to be more effective than those containing only IgG. In a previous study Berlot et al. demonstrated that early administration of ivIgGAM was associated with lower mortality rate. We studied a larger population of similar patients aiming either to confirm or not this finding considering also the subgroup of patients with septic shock by multidrug-resistant (MDR) pathogens.

**Methods:**

Adult patients with septic shock in intensive care unit (ICU) treated with ivIgGAM from August 1999 to December 2016 were retrospectively examined. Collected data included the demographic characteristics of the patients, the diagnosis at admission, SOFA, SAPS II and Murray Lung Injury Score (LIS), characteristics of the primary infection, the adequacy of antimicrobial therapy, the delay of administration of ivIgGAM from the ICU admission and the outcome at the ICU discharge. Parametric and nonparametric tests and logistic regression were used for statistic analysis.

**Results:**

During the study period 107 (30%) of the 355 patients died in ICU. Survivors received the ivIgGAM earlier than nonsurvivors (median delay 12 vs 14 h), had significantly lower SAPS II, SOFA and LIS at admission and a lower rate of MDR- and fungal-related septic shock. The appropriateness of the administration of antibiotics was similar in survivors and nonsurvivors (84 vs 79%, respectively, *p*: n.s). The delay in the administration of ivIgGAM from the admission was associated with in-ICU mortality (odds ratio per 1-h increase = 1.0055, 95% CI 1.003–1.009, *p* < 0.001), independently of SAPS II, LIS, cultures positive for MDR pathogens or fungi and onset of septic shock. Only 46 patients (14%) had septic shock due to MDR pathogens; 21 of them (46%) died in ICU. Survivors had significantly lower SAPS II, SOFA at admission and delay in administration of ivIgGAM than nonsurvivors (median delay 18 vs 66 h). Even in this subgroup the delay in the administration of ivIgGAM from the admission was associated with an increased risk of in-ICU mortality (odds ratio 1.007, 95% CI 1.0006–1.014, *p* = 0.048), independently of SAPS II.

**Conclusions:**

Earlier administration of ivIgGAM was associated with decreased risk of in-ICU mortality both in patients with septic shock caused by any pathogens and in patients with MDR-related septic shock.

**Electronic supplementary material:**

The online version of this article (10.1186/s13613-018-0466-7) contains supplementary material, which is available to authorized users.

## Background

In the last few years it became clear that septic shock can occur in two different forms: the first is characterized by an excessive pro-inflammatory response due to the interaction between the host and the infecting germ whereas the other is associated with the progressive exhaustion of both the innate and adaptive immune system; the consequent decrease in the number of B-cells and of the production of immunoglobulins (Ig) often leads to secondary infections which can negatively affect the clinical outcome [1, 2]. The role of endogenous Ig has been enlightened by a number of investigations which demonstrated that (a) the concentrations of different classes of Ig were decreased both in community- and ICU-acquired septic shock; and that (b) these findings were associated with a greater vasopressor requirement, higher incidence of ARDS and increased mortality [3–5]. The protective effects of Ig are ascribed to their pleiotropic actions, including the enhancement of bacterial and viral clearance, the decreased synthesis of several pro-inflammatory cytokines and the scavenging of apoptotic cells [6].

Despite a better knowledge of their mechanisms of action and some meta-analyses demonstrating that (a) the administration of polyclonal intravenous Ig (ivIg) was associated with an improved outcome in septic patients; and (b) this effect was more marked when the preparation used in the trial contained increased amount of IgA and IgM (12% each one) (ivIgGAM) [7–10], the previous as well as the current guidelines of the Surviving Sepsis Campaign (SSC) recommend against their use; this statement is based on either the lack of randomized clinical trials (RCT) satisfying the evidence-based medicine (EBM) standards and to the different composition of the ivIg used [11]. Independently by the SSC, not only the ivIgGAM are widely used but a new preparation containing almost double concentrations of IgM (⁓23%) and IgA (⁓21%) has been developed whose use has been associated with an improved survival in patients with community-acquired pneumonia enrolled in a recent RCT (CIGMA) [12]; noteworthy, this effect was more marked in patients with baseline elevated levels of C-reactive protein, low levels of IgM or a combination of these abnormalities, possibly indicating a subset of patients who could take the maximal advantage from this approach.

The beneficial effect of IgM could be ascribed to its pentameric structure, including the neutralization of exo- and endotoxins, the enhancement of opsonization and phagocytosis and the increased bacterial lysis obtained via the activation of the alternative pathway of the complement system [12–21]; moreover, besides these anti-infective actions, IgM molecules have also some immunomodulatory effects such as the scavenging of excessive complement factors and the blunting of the production of some sepsis mediators [22].

As in a previous study Berlot et al. [23] demonstrated that in a group of severe sepsis and septic shock patients survivors were given ivIgGAM earlier than nonsurvivors and that each 24-h delay was associated with > 2% increase in the mortality rate, we studied a much larger population of similar patients aiming either to confirm or not this finding considering also the site of infection and the responsible germs.

## Patients and methods

**Fig. 1:**
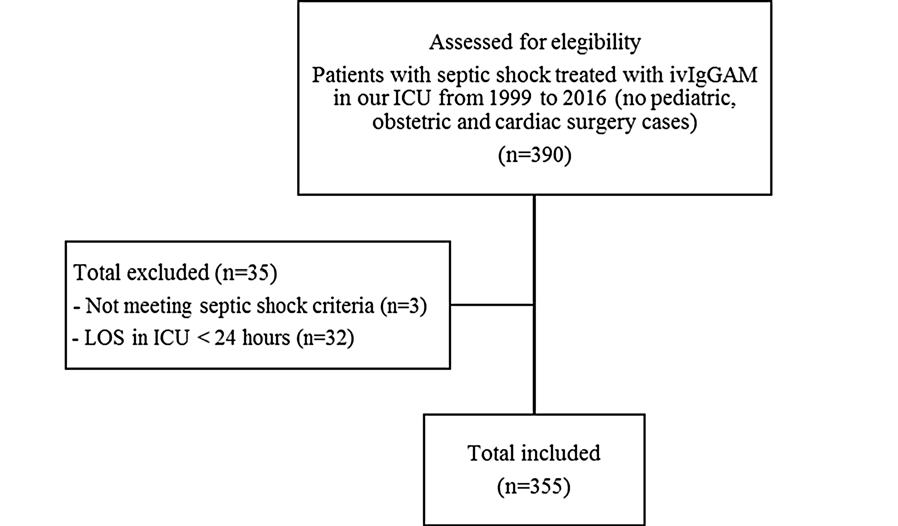
Flowchart with inclusion and exclusion criteria. ivIgGAM, intravenous IgM- and IgA-enriched immunoglobulins; ICU intensive care unit; LOS length of stay

The hospital pharmacy provided names of all patients with septic shock admitted in the intensive care unit (ICU) of the University of Trieste since August 1999 to December 2016 and treated with ivIgGAM (Pentaglobin^®^; Biotest, Dreieich, Germany). The medical records of these patients were retrospectively examined except for patients who died within 24 h from admission in ICU and patients who did not meet SEPSIS 3 criteria for septic shock [24] (see flowchart Fig. 1). As ivIgGAM are routinely used in our ICU, the study did not imply any active intervention other than the standard medical care, so that the local Ethical committee deemed the patients’ informed consent unnecessary. Critically ill adult patients with trauma, medical and surgical critical illnesses are admitted in this ICU, whereas patients with noncomplicated acute cardiac diseases and following cardiac surgery are treated in another department; pediatric and obstetric patients are admitted to another hospital.

The overall treatment was based on the SSC guidelines [11]. In particular in our ICU, the antimicrobial therapy was and is currently based on local guidelines which are periodically updated according to the microbiological reports; low-dose steroids at a dose not exceeding 200 mg/day were administered in patients not responding to fluids and vasoactive drugs. According to the international guidelines, fluids were administered respecting 3-h bundles. As an adjunctive treatment, every patient received the selective digestive tract decontamination (SDD) for the whole length of stay in ICU associated with iv cefotaxime for the initial 3 days. Oral and rectal swabs were obtained at admission and twice a week subsequently. Patients with a life expectancy < 3 months, with known immune depression (i.e., AIDS, acute leukemia.) and/or treated with immunosuppressant agents were not included in the study.

Septic shock was considered to be caused by MDR bacteria when cultures obtained within 12 h from the diagnosis were positive for at least one of the following: methicillin-resistant *Staphylococcus aureus* (MRSA), methicillin-resistant *Staphylococcus epidermidis* (MRSE), extended-spectrum beta-lactamase (ESBL)-producing *Enterococcus faecium,* vancomycin-resistant Enterococci (VRE), multidrug-resistant *Acinetobacter,* ESBL-producing Enterobacteriaceae, carbapenem-resistant Enterobacteriaceae and multidrug-resistant *Pseudomonas.*

The ivIgGAM were administered as adjunctive therapy at the dose of 250 mg/kg/day; the infusion lasted 10 h and was repeated for 3 days (total dose 750 mg/kg), according to the manufacturer’s indications.

Collected data included the demographic characteristics of the patients (age and sex), the type of admission (surgical or medical admission), the diagnosis at admission, the SOFA score calculated the first day of administration of ivIgGAM (SOFA D1), the SAPS II score, the Murray Lung Injury Score (LIS), the site of occurrence of septic shock, which was considered ICU-acquired when it occurred ≥ 48 h after the admission, the primary site of infection, the antibiotics administered and their adequacy, the microorganisms isolated within 12 h from the diagnosis, the interval elapsing from the ICU admission and the first administration of ivIgGAM, the LOS_ICU_ and the outcome at the ICU discharge. The goal of the study was to estimate the effect of the timing of administration of ivIgGAM on in-ICU mortality in patients with septic shock caused either by antibiotic-sensible or MDR pathogens. Since it was not aimed to compare the effectiveness of different treatments, it was not necessary to identify a control group.

To assess these relationships, initial descriptive comparisons between survivors and nonsurvivors at discharge from ICU were carried out. Continuous variables were described by median and interquartile range and compared with the Wilcoxon–Mann–Whitney test (and the ANOVA test in case of ivIgGAM delay, in order to compare also the means), whereas categorical variables were described by absolute and relative frequencies and compared with the χ^2^ test and the *z* score for proportions. Then, univariable logistic regression models were estimated for each parameter and subsequently a multivariable logistic regression model was build; we selected the final subset of predictors by means of a backward conditional step-wise procedure starting from the list of variables that resulted to be significantly associated with the outcome at univariable analyses. The Hosmer–Lemeshow test was used to assess the goodness of fit of estimated models. A *p* value < 0.05 was considered significant. Finally, the prediction accuracy of the multivariable regression model was evaluated by means of a Receiver Operating Characteristic (ROC) curve and the Area Under the Curve (AUC) was compared with that of the ROC curves of any of the parameters used in the model using the De Long test. The same analyses were applied to the subset of patients with septic shock caused by MDR bacteria, considering at univariable analysis a significant *p* value < 0.10 due to the small sample size, but maintaining at multivariable analysis a cutoff of *p* value < 0.05. To evaluate whether the effect of the early administration of ivIgGAM on the outcome had significant interactions with some of the most relevant patient’s characteristics, we performed further subgroup analyses. Variables considered were: the primary site of infection, the result of the cultures and the SOFA score, as a patient severity index.

All statistical computations were calculated using the R statistical package, software version 3.3.3.

## Results

During the considered period 355 patients met the inclusion criteria: 107 of them (30%) died in ICU. All patients received the first dose of empirical antimicrobial therapy within 2 h from septic shock diagnosis. Cultures were positive in 264 patients (74%) (in particular 201 cases reported positive blood cultures) and negative in 58 (17%). In 33 patients (9%) cultures within 12 h from the diagnosis were not obtained. The most frequently isolated microorganisms were: Escherichia coli (91 cases), Enterococcus faecium (36), Klebsiella pneumoniae (27), methicillin-sensitive Staphylococcus aureus (26), Enterococcus faecalis (25), MRSA (22), Pseudomonas aeruginosa (18). The median of the delay in the administration of ivIgGAM from the admission in ICU was 12 h (interquartile range 3-39). Even if the median value of delay was not significantly different between survivors and nonsurvivors at the Wilcoxon–Mann–Whitney test, the mean value was different (respectively, 36 vs 66 h, *p* = 0.002, ANOVA test) indicating a higher mean delay in the nonsurvivor group. The survivors had significantly lower SAPS II, SOFA and LIS score at admission and a lower rate of MDR-related septic shock. The distribution per year of enrollment was similar in the two groups (Table 1). The univariable logistic regression showed a statistically significant association between the timing in the administration of ivIgGAM and the outcome (Table 2). The other variables significantly related to a higher risk of dying in ICU were higher SAPS II, SOFA D1 and LIS and cultures positive for MDR bacteria or fungi. We verified that the year of enrollment was not a confounding factor because this variable did not influence the mortality (Table 2).

**Table 1:** Characteristics of the studied patients subdivided into Survivors and Nonsurvivors at discharge from ICU

	All patients (355)	Survivors (248)	Nonsurvivors (107)	*p* Value
Age (years)	68 (57-74)	68 (57–74)	70 (58–76)	0.27
Sex				0.197
Female	151 (42.54%)	111 (44.8%)	40 (37.4%)	
Male	204 (57.46%)	137 (55.2%)	67 (62.6%)	
SAPS II	53 (45–62)	51 (43–58)	57 (49–69)	< 0.001
SOFA D1	11 (9–13)	10 (8–12)	12 (11–14)	< 0.001
Murray Lung Injury Score	3 (1–5)	3 (1–5)	4 (3–6)	< 0.001
Type of admission				0.052
Surgical	264 (74.8%)	192 (77.7%)	77 (67.9%)	
Medical	89 (25.2%)	55 (22.3%)	34 (32.1%)	
Onset of septic shock				0.08
Extra ICU	265 (75.07%)	179 (72.5%)	86 (81.1%)	
Intra-ICU	88 (24.93%)	68 (27.5%)	20 (18.9%)	
Primary site of infection				< 0.001
Abdomen	198 (55.77%)	150 (60.5%)	48 (44.9%)	*p* = 0.006
Skin	27 (7.61%)	15 (6.0%)	12 (11.2%)	
Bloodstream	11 (3.1%)	6 (2.4%)	5 (4.7%)	
Not identified	19 (5.35%)	11 (4.4%)	8 (7.5%)	
Lungs	64 (18.03%)	33 (13.3%)	31 (29.0%)	*p* < 0.001
Urinary tract	31 (8.73%)	28 (11.3%)	3 (2.8%)	*p* = 0.018
Central nervous system	5 (1.41%)	5 (2.0%)	0 (0.0%)	
Adequacy of antimicrobial therapy				0.26
No	45 (17%)	28 (15%)	17 (21%)	
Yes	219 (83%)	155 (84%)	64 (79%)	
MDR bacteria				0.01
Absent	277 (85.76%)	202 (89%)	75 (78.1%)	
Present	46 (14.24%)	25 (11%)	21 (21.9%)	
Fungi				0.014
Absent	262 (81.11%)	192 (84.6%)	70 (72.9%)	
Present	61 (18.89%)	35 (15.4%)	26 (27.1%)	
Length of stay (days)	10 (6–17)	11 (6–17)	9 (3–17)	0.019
Year of enrollment	2011 (2008–2013)	2011 (2008–2013)	2011 (2008–2013)	0.57
Delay in ivIgGAM administration from admission in ICU (hours)	12 (3–39)	12 (3–33)	14 (3–66)	0.22

Variables are medians (interquartile range) or absolute frequencies (relative frequencies)

*ivIgGAM* Intravenous IgM- and IgA-enriched immunoglobulins; *ICU* intensive care unit; *MDR* multidrug resistant; *SAPS II* Simplified Acute Physiology Score; *SOFA D1*, sequential organ failure assessment calculated the first day of administration of ivIgGAM

**Table 2:** Univariable logistic regression analyses of risk factors for in-ICU mortality

	OR	95% CI	*p* Value
Age	1.004	0.98–1.02	0.62
Sex (male)	1.36	0.86–2.17	0.198
SAPS II	1.046	1.028–1.065	< 0.001
SOFA D1	1.24	1.15–1.35	< 0.001
Murray Lung Injury Score	1.14	1.04–1.24	0.003
Onset of septic shock (Intra-ICU)	0.61	0.34–1.06	0.08
Type of admission (Surgical)	0.607	0.37–1.006	0.053
MDR bacteria	2.26	1.18–4.28	0.01
Fungi	2.037	1.14–3.62	0.01
Year of enrollment	0.97	0.91–1.023	0.253
Adequacy of antimicrobial therapy	0.68	0.348–1.328	0.259
Delay in ivIgGAM administration from admission in ICU	1.0039	1.0013–1.0066	0.0035

*ivIgGAM* Intravenous IgM- and IgA-enriched immunoglobulins; *ICU* intensive care unit; *MDR* multidrug resistant; *SAPS II* Simplified Acute Physiology Score; *SOFA D1* sequential organ failure assessment calculated the first day of administration of ivIgGAM

**Fig. 2:**
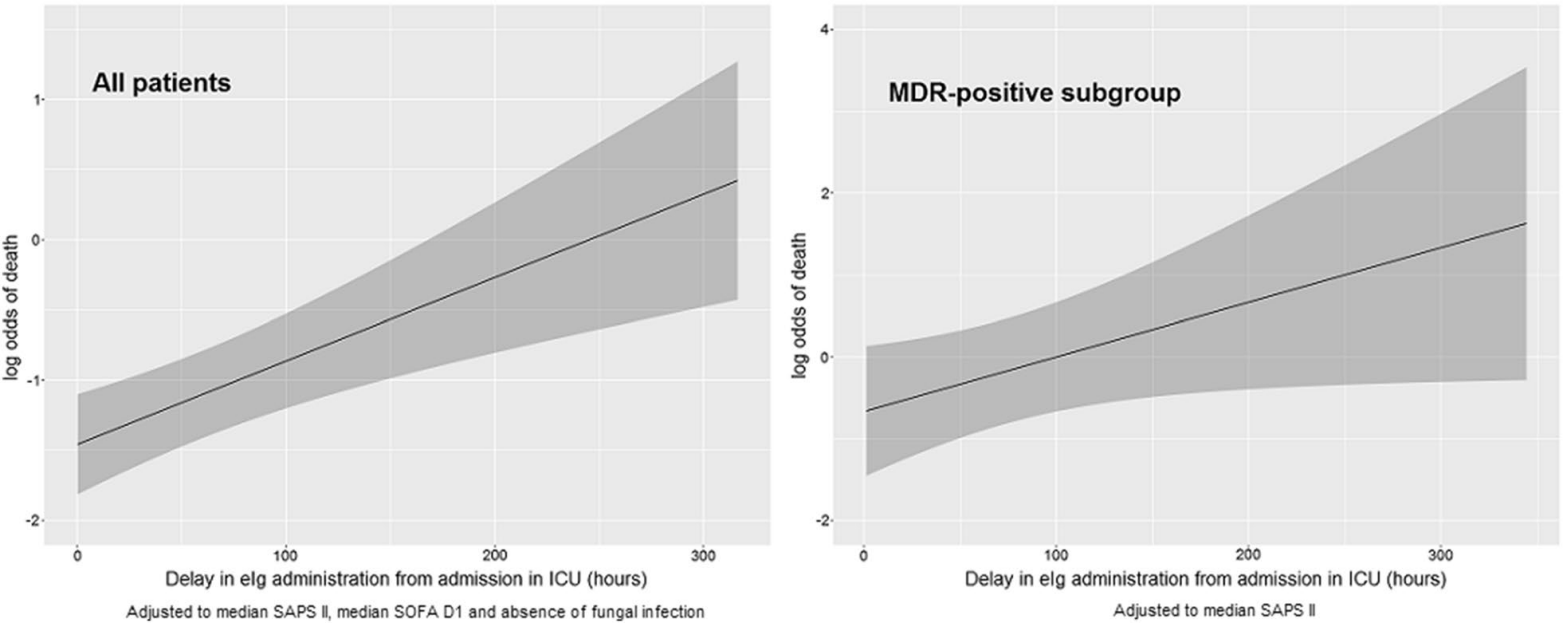
Logit function from the multivariable logistic regression model in the group of all patients (right) showing the effect of delay in administration of IgM-enriched immunoglobulins on the probability of in-ICU death (solid line) with 95% confidence interval (gray area) adjusted to median value of SAPS II, SOFA D1 and without fungal infection. Comparison with logit functions from the multivariable logistic regression model for MDR-positive patients adjusted for median value of SAPS II (left). ivIgGAM, intravenous IgM- and IgA-enriched immunoglobulins; ICU, intensive care unit; MDR, multidrug resistant; SAPS II, Simplified Acute Physiology Score; SOFA D1, sequential organ failure assessment calculated the first day of administration of ivIgGAM

The multivariable analysis showed that the association between the delay in the administration of ivIgGAM and the outcome was independent from the other variables taken into account (for each 24-hour increase adjusted OR 1.15, CI 95% 1.05-1.27, *p* = 0.0005) (Table 3), Hosmer and Lemeshow test *p* value = 0.20. Put differently, in a patient with median SAPS II and median SOFA D1, without fungal infections, a 24-hour delay in ivIgGAM administration from the admission resulted in roughly a 2% increase in the probability of dying during his ICU stay. The estimated effect of delay in administration of ivIgGAM on the probability of in-ICU death is represented in Fig. 2 using the corresponding logit function estimated from the multivariable logistic regression model, adjusted to median value of SAPS II, SOFA D1 and without fungal infection (these values were chosen as the most representative values in this population). As shown in Fig. 2, since the relationship between the delay in drug administration and the risk of death in ICU appeared linear, it is not possible to identify an optimal cutoff, as survival progressively increases with the precociousness of drug administration.

**Table 3:** Multivariable logistic regression analysis of risk factors for in-ICU mortality

	OR	95% CI	*p* Value
SAPS II	1.04	1.018–1.06	< 0.001
SOFA D1	1.15	1.052–1.27	0.003
Fungi	2.21	1.17–4.18	0.015
Delay in ivIgGAM administration from admission in ICU	1.006	1.003–1.009	< 0.001

*ivIgGAM* Intravenous IgM- and IgA-enriched immunoglobulins; *ICU* intensive care unit; *SAPS II* Simplified Acute Physiology Score; *SOFA D1* sequential organ failure assessment calculated the first day of administration of ivIgGAM

**Fig. 3:**
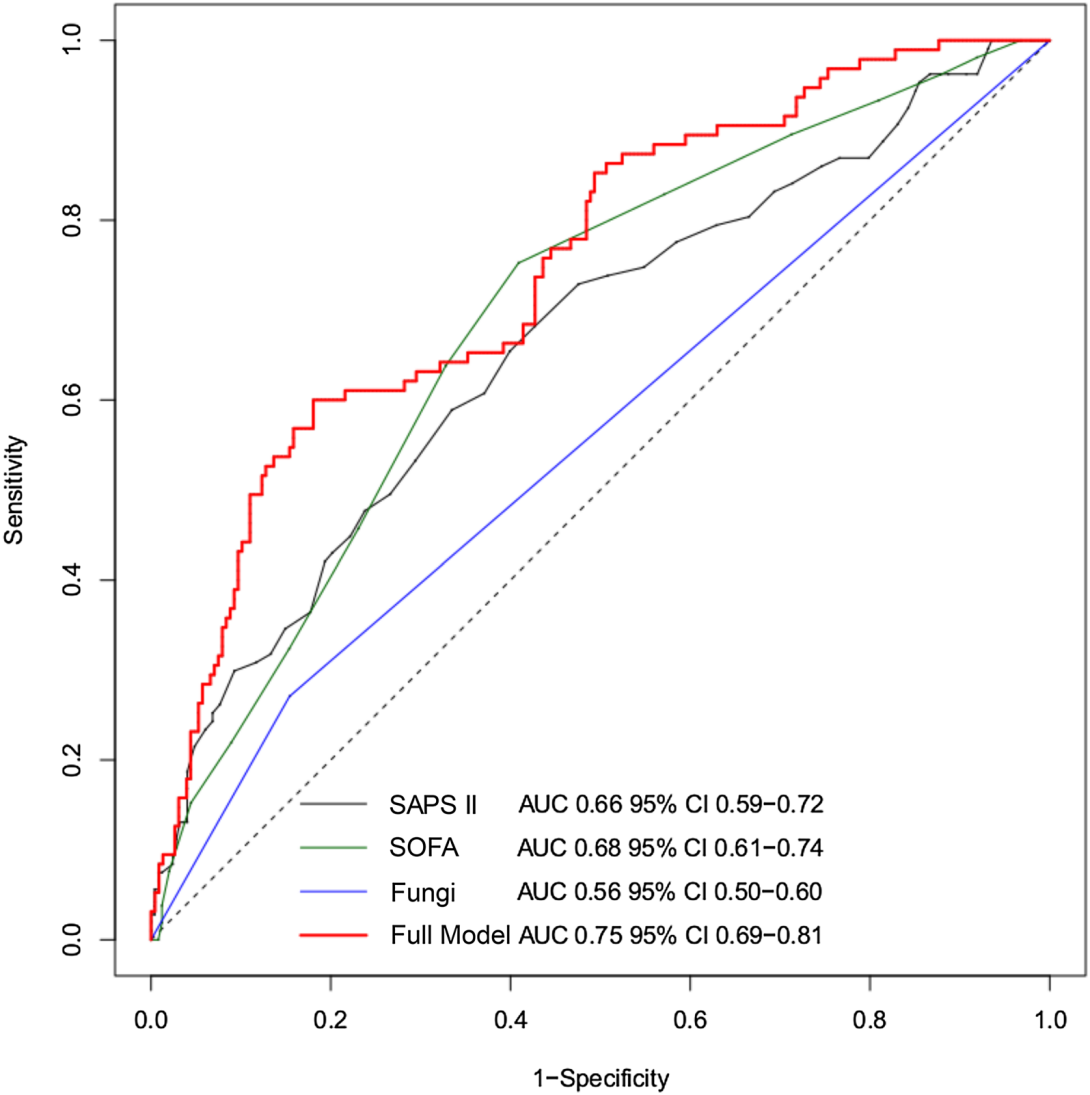
ROC curves comparing the predictive accuracy of the multivariable regression model (red curve) for all patients with each parameter used in the model: SAPS II (gray curve), SOFA D1 (green curve) and fungal infection (blue curve). SAPS II, Simplified Acute Physiology Score; SOFA, sequential organ failure assessment calculated the first day of administration of ivIgGAM

The ROC curve obtained from the model showed an AUC of 0.75 with 95% CI 0.69–0.81, significantly higher than the univariable logistic models considering SAPS II, SOFA D1 and fungal infection (*p* < 0.001) (Fig. 3).

During the study period only 46 patients (14%) met the diagnosis of septic shock due to MDR bacteria and 21 of them (46%) died in ICU (Additional file 1). The most frequently isolated MDR microorganisms were: MRSA (45.6%) and Escherichia coli ESBL (21.7%). In this group of patients also an earlier administration of ivIgGAM was associated with a better outcome (see Additional file 1).

Finally, we evaluated the effect of the timing of administration of ivIgGAM on death in ICU in different subgroups. In the subgroup with pulmonary infection (64 patients), univariable analysis did not show a statistically significant association between delay in ivIgGAM administration and mortality (*p* = 0.557). In the remaining cases the correlation was significant (*p* = 0.001). We also analyzed the subgroup of patients with abdominal infection (198 patients): in this group the relationship between the delay of ivIgGAM administration and the risk of death in ICU was statistically significant (*p* = 0.008).

Considering the results of the cultures, the sample was divided into two subgroups: patients with positive cultures within 12 h from the diagnosis of septic shock (264) and patients with negative cultures (58). In this analysis, patients in whom the cultures were not performed within 12 h (33) were not taken into consideration. In the subgroup of patients with positive cultures, the delay in administration of ivIgGAM from admission in ICU remained significantly correlated with the risk of death at both univariable (*p* = 0.001) and multivariable analysis (*p* < 0.001). In the subgroup of patients with negative cultures, this correlation was not confirmed (*p* = 0.994), but the small size of the sample should be taken into account.

To assess if the relationship between the timing of ivIgGAM administration and death in ICU depended on severity, the sample was divided into two subgroups according to the median value of the SOFA score of the first day of administration of ivIgGAM (SOFA score median value = 11, see Table 1).

At univariable analyses, the relationship was less statistically significant in the subgroup with SOFA score greater than the median (OR 1.004, *p* = 0.05), while it was highly significant in the subgroup with SOFA score lower than the median (OR 1.005, *p* = 0.01). At multivariable analyses the significance of the effect of early ivIgGAM administration was similar in the two subgroups (in both cases, *p* <= 0.02), even if the estimated effect was stronger in the less severe patients (see Additional file 1).

No adverse effects attributable to the ivIgGAM were observed.

## Discussion

In septic shock the dysregulation of both arms of the immune system accounts for both the initial hyperinflammatory phase and for the subsequent shift toward a more advanced stage in which an immunoparalysis can dominate the clinical course, leading to secondary infections by MDR germs and to the reactivation of latent viruses [1, 25]. This cycle can recur several times during the ICU stay, thus leading to a chronic critical illness, which is rather common among elderly patients affected by preexistent co-morbidities as well as in debilitated subjects of all ages [26, 27].

Currently, steroids and ivIg are the only immunomodulatory agents available, pending the results of several clinical trials with more innovative agents [28, 29]. However, it appears that what can be relatively beneficial in the initial stage of sepsis and septic shock, such as steroids, could be harmful in the following one [1, 29]; conversely, the ivIgGAM are suitable both in its early and in the advanced phase, due to their combined antibacterial and immunomodulatory properties [14–21]. The biological rationale for their administration can be found in the results of some studies demonstrating that not only low levels of IgG, IgM and IgA measured at the admission but also their failed increase during the ICU stay were associated with both the transition from severe sepsis to septic shock and with a reduced survival [30–32]; yet, despite these evidences, the role of ivIgGAM in the treatment of septic shock remains controversial notwithstanding their use began several decades ago.

Basically, supporters of ivIgGAM claim that (a) their dual action on the immune response make them valuable throughout the whole clinical course of sepsis and septic shock and that (b) their positive effect on the outcome has been confirmed by a number of meta-analyses [7–10]. On the other front, opponents reply that the clinical studies published so far suffer from such an elevated heterogeneity of patients and of preparations used, so that it is impossible to draw definite conclusion from their results [13]. Despite these biases, the ivIgGAM are widely used and the German guidelines for the treatment of sepsis [33] suggest considering their administration in septic shock patients while they discourage the use of ivIg. Therefore, it appears that more data about the clinical use of ivIgGAM are keenly needed. The results of this study not only confirm some investigations but also add further information.

First, although there was a small difference in the timing of administration of ivIgGAM between S and NS, their early administration influenced the outcome; actually, a time effect has been demonstrated previously by Berlot et al. [23] who reported a 3% increase in mortality for every 24 h of delay and by Cavazzuti et al. [34] who demonstrated a reduced mortality rate in septic shock patients treated with ivIgGAM within 24 h after shock onset. This finding recalls what has been reported in other investigations, which demonstrated an increase in mortality for each hour of delay in the administration of antibiotic [35–37]. The positive effect of ivIgGAM on the outcome persisted across the whole period of the study and was independent of other variations of the overall treatment. As shown by subgroup analyses, the benefit of early ivIgGAM administration appears to be more intense in clinically less severe patients, as already reported in the literature [38] and greater for abdominal primary site of infection. This time effect appears to be independent from the adequacy of the antibiotic therapy since this latter variable did not differ among the two groups.

Second, this “time window effect” was present also in the group of patients whose infections were caused by a wide array of MDR pathogens. Although the timing of ivIgGAM administration was not specifically addressed in their studies, also Busani [39] and Giamarellos [40] demonstrated an increased survival in MDR germs-related septic shock treated with ivIgGAM as compared with the control group. However, it is worthwhile to remark that (a) the rate of MDR infections in our population was considerably lower than the one reported in these studies and that (b) since only 1 out of 3 cases of MDR-related septic shock was ICU-acquired, it is possible that the administration of ivIgGAM in patients already admitted to the ICU prevented their colonization and subsequent infection with these germs. Then, it is hypothesizable that in the acute phase the ivIgGAM could act by increasing the bacterial clearance and reducing the hyperinflammatory reaction while in the later one they could contribute to restore the compromised immune capabilities by replenishing the IgM and IgA stores.

Our study has some limitations, including (a) the absence of the EBM criteria such as the randomization of the patients and their prolonged time of enrollment and lack of validation of the results in an independent cohort; however, as stated by some authors [29, 41] the past issues of the SSC guidelines contained several recommendations derived from the results of EBM- based RCTs that were not confirmed by subsequent studies and were removed later on; (b) the lack of a biological marker, such as the measurement of the blood levels of CRP and PCT and/or IgG, IgM and IgA suited to trigger the administration of ivIgGAM; actually, this reflects the real-world scenario because the results of these and other tests evaluating the immune capability are not universally available and, more important, what is a “safe” concentration of endogenous IgM and IgA in septic shock patients is not clear [13]; (c) due to the wide time span of the study, we could not establish precisely the rate of the appropriate antimicrobial treatment; however, as antibiotics were given according to the yearly updated hospital guidelines based on the local microbiological findings, we guess that it was evenly distributed among survivors and deceased patients.

## Conclusions

In our experience, the administration of ivIgGAM was safe and well tolerated. Overall, survivors were treated earlier than nonsurvivors and this finding was present also in patients with MDR-associated septic shock, a specific sepsis population with high mortality risk.

In the perspective of personalized medicine, further studies and particularly RCTs are needed to identify the characteristics of the best septic shock candidate to receive ivIgGAM as adjunctive treatment. Our study contributes by showing the potential effect of the time factor on the effectiveness of ivIgGAM.

## Additional file

**Additional file 1.** Adjustment of analyses for severity and periods of enrollment and subgroup analyses of MDR-related septic shock.

## Abbreviations

AUC: area under the curve
ICU: intensive care unit
ivIg: polyclonal intravenous immunoglobulins
ivIgGAM: intravenous IgM- and IgA-enriched immunoglobulins
Ig: immunoglobulins
LIS: Murray Lung Injury Score
MDR: multidrug resistant
MRSA: methicillin-resistant *Staphylococcus aureus*
MRSE: methicillin-resistant *Staphylococcus epidermidis*
SBL: extended-spectrum beta-lactamase
VRE: vancomycin-resistant Enterococci
SAPS II: Simplified Acute Physiology Score
SDD: selective digestive tract decontamination
SOFA: sequential organ failure assessment
ROC: receiver operating characteristic
