# Correction to: Effects of the timing of administration of IgM- and IgA-enriched intravenous polyclonal immunoglobulins on the outcome of septic shock patients

**DOI:** 10.1186/s13613-019-0504-0

**Published:** 2019-03-05

**Authors:** Giorgio Berlot, Michele Claudio Vassallo, Nicola Busetto, Margarita Nieto Yabar, Tatiana Istrati, Silvia Baronio, Giada Quarantotto, Mattia Bixio, Giulia Barbati, Roberto Dattola, Irene Longo, Antonino Chillemi, Alice Scamperle, Fulvio Iscra, Ariella Tomasini

**Affiliations:** 1 Department of Anesthesia, Resuscitation and Pain Therapy, Cattinara Hospital, University of Trieste, Strada di Fiume 447, 34149 Trieste, Italy; 2 Department of Anesthesia and Intensive Care, San Martino Hospital, Largo Rosanna Benzi 10, 16132 Genoa, Italy; 3 Biostatistics Unit, Department of Medicine, Surgery and Health Sciences, Cattinara Hospital, University of Trieste, Strada di Fiume 447, 34149 Trieste, Italy; 4 Department of Cardiac Anesthesia and Intensive Care, ASST Fatebenefratelli-Sacco-Buzzi, Via G.B. Grassi 74, 20157 Milan, Italy

## Correction to: Ann. Intensive Care (2018) 8:122 10.1186/s13613-018-0466-7

Following publication of the original article [1], we have been notified that the tagging of the author name was done incorrectly in the XML version of the paper. The correct given name is Michele Claudio, and the family name is Vassallo.

The online and pdf versions of this paper are not affected by the change.

The publisher apologizes for any inconvenience caused by this error.

## References

[1] Berlot G (2018). Ann. Intensive Care.

