# Supplementary material for: Effects of the timing of administration of IgM- and IgA-enriched intravenous polyclonal immunoglobulins on the outcome of septic shock patients
Source: Ann Intensive Care. 2018 Dec 10;8:122. doi: 10.1186/s13613-018-0466-7 (PMC6288102; doi:10.1186/s13613-018-0466-7)

**Supplemental Materials**

**Subgroup analyses based on severity (according to SOFA score)**

Univariable analyses:

**Table I.** Subgroup with SOFA score higher than median value (11)

|  | **OR** | **95% CI** | **p-value** |
| --- | --- | --- | --- |
| Delay in ivIgGAM administration from admission in ICU | 1.004 | 1.00-1.008 | 0.054 |

**Table II.** Subgroup with SOFA score lower than median value (11)

|  | **OR** | **95% CI** | **p-value** |
| --- | --- | --- | --- |
| Delay in ivIgGAM administration from admission in ICU | 1.005 | 1.001-1.009 | 0.012 |

Multivariable analyses:

**Table III.** Subgroup with SOFA score higher than median value (11)

|  | **OR** | **95% CI** | **p-value** |
| --- | --- | --- | --- |
| SAPS2 | 1.046 | 1.018-1.075 | 0.001 |
| SOFA D1 | 0.969 | 0.807-1.163 | 0.733 |
| Fungi | 2.734 | 1.212-6.167 | 0.015 |
| Delay in ivIgGAM administration from admission in ICU | 1.005 | 1.001-1.010 | 0.016 |

**Table IV.** Subgroup with SOFA score lower than median value (11)

|  | **OR** | **95% CI** | **p-value** |
| --- | --- | --- | --- |
| SAPS2 | 1.035 | 0.996-1.076 | 0.081 |
| SOFA D1 | 1.188 | 0.885-1.595 | 0.252 |
| Fungi | 1.106 | 0.325-3.761 | 0.871 |
| Delay in ivIgGAM administration from admission in ICU | 1.007 | 1.003-1.011 | 0.002 |

**Adjustment of multivariable analysis for periods**

Patients were subdivided into 4 subgroups according to the period of enrollment. We considered periods lasting 5 years (as the Reviewer suggested), with the exception of the last subgroup involving patients enrolled from 2014 to 2016 (when the data collection ended). The period of enrollment was not significantly related to the risk of death in ICU both at univariable and multivariable analyses (p=0.28 and p=0.49 respectively).

**Table V.** Absolute and relative frequencies of patients according to 5-years periods of enrollment

|  | **Absolute frequency (n)** | **Relative frequency (%)** |
| --- | --- | --- |
| 1999 - 2003 | 22 | 6.2 |
| 2004 - 2008 | 77 | 21.7 |
| 2009 - 2013 | 169 | 47.6 |
| 2014 - 2016 | 87 | 24.5 |
| Total | 355 | 100.0 |

**Table VI.** Univariable logistic analysis of risk of death in ICU according to 5-years periods of enrollment

|  | **OR** | **95% CI** | **p-value** |
| --- | --- | --- | --- |
| Year of enrollment in 5-years periods | 0.863 | 0.660-1.130 | 0.285 |

**Table VII.** Multivariable logistic analysis of risk of death in ICU according to 5-years periods of enrollment

|  | **OR** | **95% CI** | **p-value** |
| --- | --- | --- | --- |
| SAPS2 | 1.041 | 1.019-1.063 | 0.000 |
| SOFA D1 | 1.155 | 1.052-1.269 | 0.003 |
| Fungi | 2.263 | 1.189-4.307 | 0.013 |
| Delay in ivIgGAM administration from admission in ICU | 1.006 | 1.003-1.010 | 0.000 |
| Year of enrollment in 5-years periods | 1.131 | 0.796-1.607 | 0.493 |

**MDR-related septic shock subgroup**

In the subgroup of patients with septic shock caused by MDR strains the median delay in administration of ivIgGAM from the admission was 28 hours (IQR 6-85 hours). The comparison between S and NS showed significant differences in delay in administration of ivIgGAM from the admission (p=0.09), SAPS II (p=0.047) and SOFA D1 (p=0.098) which were higher in the NS group. At the univariable regression analysis SAPS II was the only variable associated to an increased risk of in-ICU mortality (Supplemental Material – Table II). At multivariable regression analysis, the delay in the administration of ivIgGAM from the admission was associated to an increased risk of in-ICU mortality independently of SAPS II (for 24 hours increase OR=1.17, CI 95%=1.0009-1.38, p=0.048). (Supplemental Material – Table III), Hosmer and Lemeshow test p-value = 0.19. In a patient with median SAPS II, 24 hours delay in the ivIgGAM administration resulted in around 3.5% increase in the probability of dying during the ICU stay.

The ROC curve evaluating the accuracy of the multivariable logistic model in this subgroup had an AUC=0.76 (95% CI=0.61–0.90). (Supplemental Material – Figure).

**Table VIII**. Characteristics of patients with septic shock caused by MDR bacteria subdivided into Survivors and Nonsurvivors at discharge from ICU

|  | **All patients (46)** | **Survivors (25)** | **Nonsurvivors (21)** | **p value** |
| --- | --- | --- | --- | --- |
| Age (years) | 69 (57-77) | 70 (59-77) | 67 (55-77) | 0.52 |
| Sex |  |  |  | 0.923 |
| Female | 15 (32.6%) | 8 (32%) | 7 (33.3%) |  |
| Male | 31 (67.4%) | 17 (68%) | 14 (66.7%) |  |
| SAPS II | 56 (48-64) | 52 (48-59) | 60 (54-67) | 0.047 |
| SOFA D1 | 12 (10-14) | 11 (9-13) | 12 (11-14) | 0.098 |
| Murray Lung Injury Score | 4 (2-7) | 3 (2-6) | 5 (2-7) | 0.42 |
| Type of admission |  |  |  | 0.484 |
| Surgical | 33 (71.7%) | 19 (76%) | 14 (66.7%) |  |
| Medical | 13 (28.3%) | 6 (24%) | 7 (33.3%) |  |
| Onset of septic shock |  |  |  | 0.42 |
| Extra ICU | 30 (65.2%) | 15 (60%) | 15 (71.4%) |  |
| Intra ICU | 16 (34.8%) | 10 (40%) | 6 (28.6%) |  |
| Primary site of infection |  |  |  | 0.2 |
| Abdomen | 25 (54.3%) | 16 (64%) | 9 (42.9%) |  |
| Skin | 4 (8.7%) | 2 (8%) | 2 (9.5%) |  |
| Bloodstream | 2 (4.3%) | 1 (4%) | 1 (4.8%) |  |
| Not identified | 0 (0.0%) | 0 (0%) | 0 (0.0%) |  |
| Lungs | 13 (28.3%) | 4 (16%) | 9 42.9%) |  |
| Urinary tract | 2 (4.3%) | 2 (8%) | 0 (0.0%) |  |
| Central nervous system | 0 (0.0%) | 0 (0.0%) | 0 (0.0%) |  |
| Adequacy of antimicrobial therapy |  |  |  | 0.35 |
| No | 25 (54%) | 12 (48%) | 13 (62%) |  |
| Yes | 21 (46%) | 13 (52%) | 8 (38%) |  |
| Gram-negative | 20 (43.5%) | 9 (36%) | 11 (52.4%) | 0.26 |
| Gram-positive | 26 (56.5%) | 16 (64%) | 10 (47.6%) |  |
| Length of stay (days) | 8 (7-14) | 9 (7-14) | 7 (6-15) | 0.35 |
| Delay in ivIgGAM administration from admission in ICU (hours) | 28 (6-85) | 18 (6-38) | 66 (15-166) | 0.09 |
| Variables are medians (interquartile range) or absolute frequencies (relative frequencies).  ivIgGAM, intravenous IgM- and IgA-enriched immunoglobulins; ICU, Intensive Care Unit; SAPS II, Simplified Acute Physiology Score; SOFA D1, Sequential Organ Failure Assessment calculated the first day of administration of ivIgGAM. | | | | |

|  | **OR** | **95% CI** | **p value** |
| --- | --- | --- | --- |
| Age | 0.94 | 0.27-3.3 | 0.923 |
| Sex (Male) | 0.76 | 0.22-2.60 | 0.66 |
| SOFA D1 | 1.19 | 0.97-1.51 | 0.12 |
| SAPS II | 1.057 | 1.001-1.125 | 0.058 |
| Murray Lung Injury Score | 1.12 | 0.9-1.4 | 0.31 |
| Onset of septic shock (Intra-ICU) | 0.6 | 0.17-2.04 | 0.42 |
| Type of admission (Surgical) | 0.63 | 0.17-2.29 | 0.48 |
| Gram (Gram-negative) | 1.95 | 0.6-6.56 | 0.27 |
| Adequacy of antimicrobial therapy | 0.56 | 0.17-1.83 | 0.35 |
| Delay in ivIgGAM administration from admission in ICU | 1.0048 | 0.999-1.012 | 0.10 |
| ivIgGAM, intravenous IgM- and IgA-enriched immunoglobulins; ICU, Intensive Care Unit; SAPS II, Simplified Acute Physiology Score; SOFA D1, Sequential Organ Failure Assessment calculated the first day of administration of ivIgGAM. | | | |

**Table IX.** Univariable logistic analyses of risk factors for in-ICU mortality in patients with septic shock caused by MDR bacteria

**Table X**. Multivariable logistic analysis of risk factors for in-ICU mortality in patients with septic shock caused by MDR bacteria

|  | **OR** | **95% CI** | **p value** |
| --- | --- | --- | --- |
| SAPS II | 1.073 | 1.0125-1.148 | 0.025 |
| Delay in ivIgGAM administration from admission in ICU | 1.0067 | 1.0006-1.014 | 0.048 |
| ivIgGAM, intravenous IgM- and IgA-enriched immunoglobulins; ICU, Intensive Care Unit; SAPS II, Simplified Acute Physiology Score. | | | |

**Figure.** ROC curve comparing the predictive accuracy of the multivariable regression model (red curve) for patients with septic shock by MDR bacteria with each parameter used in the model: SAPS II (black curve). SAPS, Simplified Acute Physiology Score


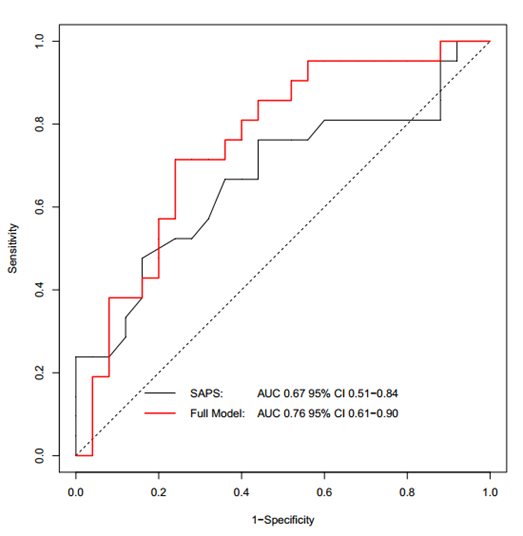

Supplement: Supplementary file 1 — Additional file 1. Adjustment of analyses for severity and periods of enrollment and subgroup analyses of MDR-related septic shock. [file 13613_2018_466_MOESM1_ESM.docx]
